# Selective synthesis of pure cobalt disulfide on reduced graphene oxide sheets and its high electrocatalytic activity for hydrogen evolution reaction

**DOI:** 10.1186/s40580-016-0066-x

**Published:** 2016-01-27

**Authors:** Seongjoon Ahn, Jieun Yang, Hyunseob Lim, Hyeon Suk Shin

**Affiliations:** 1 Department of Chemistry, Ulsan National Institute of Science and Technology (UNIST), UNIST-gil 50, Ulsan, 689-798 Republic of Korea; 2 Department of Energy Engineering, Ulsan National Institute of Science and Technology (UNIST), UNIST-gil 50, Ulsan, 689-798 Republic of Korea; 3 Low Dimensional Carbon Materials, Ulsan National Institute of Science and Technology (UNIST), UNIST-gil 50, Ulsan, 689-798 Republic of Korea; 4 Center for Multidimensional Carbon Materials, Institute of Basic Science, Ulsan National Institute of Science and Technology (UNIST), UNIST-gil 50, Ulsan, 689-798 Republic of Korea; 5 Department of Material Science and Engineering, Rutgers University, Piscataway, NJ 08854 USA

**Keywords:** Transition metal dichalcogenides, Cobalt disulfide, Hybrid, Electrocatalyst, Hydrogen evolution reaction

## Abstract

We synthesized single-phase CoS_2_ on a large scale by adding graphene oxide of sufficient quantity via the hydrothermal method using cobalt acetate and thioacetamide as precursors; this produced the hybrid of CoS_2_ with reduced graphene oxide which exhibited high electrocatalytic activity in the hydrogen evolution reaction.

**Electronic supplementary material:**

The online version of this article (doi:10.1186/s40580-016-0066-x) contains supplementary material, which is available to authorized users.

## Introduction

Considerable efforts have been made toward synthesizing transition metal dichalcogenide (TMD) nanomaterials due to their excellent electronic, optical, and mechanical properties [1, 2]. Among the more notable TMD compounds, MoS_2_ and WS_2_, which have electrical properties that can be changed from metallic to semiconducting by varying their crystal structure and the number of layers, have been extensively studied [1]. In addition, the structure and general properties of pyrite-phase TMDs (FeS_2_, NiS_2_, and CoS_2_) have been investigated [3–6]. These materials have attracted interest for their potential applications; for example, FeS_2_ with a band gap of 0.95 eV has been used as an active layer in photovoltaic devices and NiS_2_ has been used as a Li storage material [6–9]. In particular, CoS_2_ has received considerable attention due to its metallic behavior, which makes it applicable as an electrocatalyst for oxygen reduction reactions and hydrogen evolution reactions (HERs) [3, 10, 11].

So far, MoS_2_ and WS_2_ nanostructures have been extensively explored as electrocatalysts for HER. The overpotentials of MoS_2_ and WS_2_ materials occur between −200 and −150 mV and their Tafel slopes fall in the range of 55–40 and 70–58 mV/dec, respectively [2, 12]. Recently, metallic CoS_2_ has been recognized for its potential as a viable HER catalyst like MoS_2_. An overpotential of −180 mV and a Tafel slope of 44.6 mV/dec have been measured for CoS_2_ films synthesized by gas-phase reactions [3]. CoS_2_ micro- and nanowires have overpotentials of about −100 and –70 mV, respectively, and Tafel slopes of 58.0 and 51.6 mV/dec, respectively [10]. However, CoS_2_ film is easily damaged by delamination during H_2_ evolution. Although CoS_2_ nanowires exhibited the best performance, they became delaminated within three hours. Accounting for a long-term stability, microwires have proven the most effective, despite their limited HER performance. The microstructured surface also helps to convey the H_2_ bubbles away from the electrode surface, thus maintaining the integrity of the catalyst for HER [10]. On the other hand, the strong performance of CoS_2_ in HER may result from the oxidation state of sulfur in CoS_2_, since it is known that S_2_^2−^ exhibits higher HER efficiency than S^2−^. Chang et al. prepared MoS_x_ with S_2_^2−^ on 3D Ni foam deposited with graphene layers for electrocatalytic hydrogen evolution and found that MoS_x_ with S_2_^2−^ exhibits higher catalytic activity than MoS_2_ with S^2−^ [13, 14]. Therefore, the HER activity of pure-phase CoS_2_ should be investigated since it is expected that CoS_2_ with S_2_^2−^ dimer will exhibit favorable performance in HER. As mentioned above, pure-phase CoS_2_ nanowires and microwires were obtained by the synthesis of cobalt hydroxide carbonate hydrate (Co(OH)(CO_3_)0.5·xH2O) nanowires and cobalt hydroxide (-Co(OH)_2_) microwires through a solution-based reaction followed by thermal sulfurization [10]. Additionally, pure-phase CoS_2_ film has been synthesized by sulfurization of Co film: The substrate on which Co is deposited by an e-beam evaporator is located in the tube furnace and sulfur powder is vaporized by flowing Ar gas at 550 °C [3]. All of the methods are two-step processes, which are limited in terms of mass production. Therefore, it would be worthwhile to establish a method for the large-scale synthesis of pure CoS_2_.

The hydrothermal reaction is known as a facile method for the large-scale synthesis of CoS_2_. However, producing pure-phase CoS_2_ is quite difficult because of the complex stoichiometric compositions of cobalt sulfides such as CoS_2_, CoS, Co_9_S_8_, and Co_1−x_S that form when the hydrothermal synthesis of CoS_2_ is attempted using precursors such as cobalt chloride (as cobalt precursor) and thioacetamide, sodium thiosulfate, etc. (as sulfur precursors) [15–20]. Indeed, a mixture of cobalt sulfides has been synthesized using the hydrothermal method. For example, Huang et al. found that the flower-like cobalt sulfides prepared by a solvothermal method included a mixture of CoS and Co_9_S_8_ (9 %) [15]. Qian et al. then found that as-synthesized cobalt sulfides consisted of CoS_2_, Co_9_S_8_, and Co_3_S_4_ when using cobalt chloride in toluene [16]. Furthermore, most reports employed only X-ray diffraction (XRD) for the characterization of CoS_2_ [15–20]. However, in-depth characterization using TEM and XPS as well as XRD is required for the identification of CoS_2_, due to the existence of various stoichiometric compositions of cobalt sulfides. In addition, impurities such as oxygen can occur in CoS_2_ since it is susceptible to oxidation in air [10]. Thus, further research is required to allow for the characterization and large-scale production of pure CoS_2_ with S_2_^2−^ dimer.

Herein, we report on such a large-scale synthesis of pure-phase CoS_2_ on rGO by a one-pot hydrothermal reaction using cobalt acetate and thioacetamide precursors. We demonstrate that GO concentrations of greater than 2 mg/mL are critical in supporting the synthesis of pure-phase CoS_2_ and inhibiting the oxidation of the CoS_2_ surface. Furthermore, we provide detailed structural analyses of the synthesized CoS_2_ and investigate its electrocatalytic activity in HER, thereby demonstrating a long-term stability.

## Methods

### Synthesis of GO solution

GO was prepared from graphite powder by the modified Hummers’ method.

### Hydrothermal synthesis of CoS_2_/rGO hybrid

GO solution was added to the mixture of cobalt acetate tetrahydrate (9 mmol) and thioacetamide (90 mmol) and the total volume of the solution was adjusted to 400 mL for all reactions. The solution was transferred to a 500 mL Teflon-lined stainless steel autoclave, heated up to 240 °C, and kept for 24 h. After cooling naturally, the product was filtered, washed with DI water, and dried in vacuum at 60 °C for 12 h. During the hydrothermal process, GO was converted to rGO and CoS_2_/rGO was formed.

### Characterization

The samples were characterized with field emission scanning electron microscopy (Hitachi, S4800), high-resolution transmission electron microscopy (HRTEM, JEOL JEM-2100 F with probe-Cs corrector, 200 kV), X-ray diffraction [Rikgaku RU-200 diffractometer equipped with Ni-filtered Cu Kα radiation (40 kV, 100 mA, λ = 0.15418 nm)], and X-ray photoelectron spectroscopy (K-alpha, ThermoFisherwith monochromatic Al Kα radiation as the X-ray source). The rGO concentration of samples was characterized with element analyzer (Thermo Scientific, Flash 2000).

### Electrochemical measurements

Electrocatalytic measurements were carried out using a 3-electrode cell and a 0.5 M sulfuric acid (H_2_SO_4_) electrolyte solution. A graphite rod (Sigma Aldrich) and Ag/AgCl electrode (Wonatech) were used as counter electrode and reference respectively. The reference electrode was calibrated with respect to reversible hydrogen electrode (RHE) using platinum wires as working and counter electrodes. Materials were dispersed in deionized water at 4 mg/mL and sonicated for 1 h. The ink was then drop-casted onto glassy carbon electrodes of 3 mm diameters (loading 285 µg/cm^2^) and capped by Nafion (0.5 %, 3 µL, Sigma Aldrich). Linear sweep voltammetry was performed with a 5 mV/s scan rate using a Zive SP2 potentiostat from Wonatech and the electrodes were cycled at least 40 cycles prior to any measurement. The polarization curves were iR-corrected. Electrochemical impedance spectroscopy (EIS) was performed in the frequency range from 1 MHz to 0.1 Hz.

## Results and discussion

**Fig. 1:**
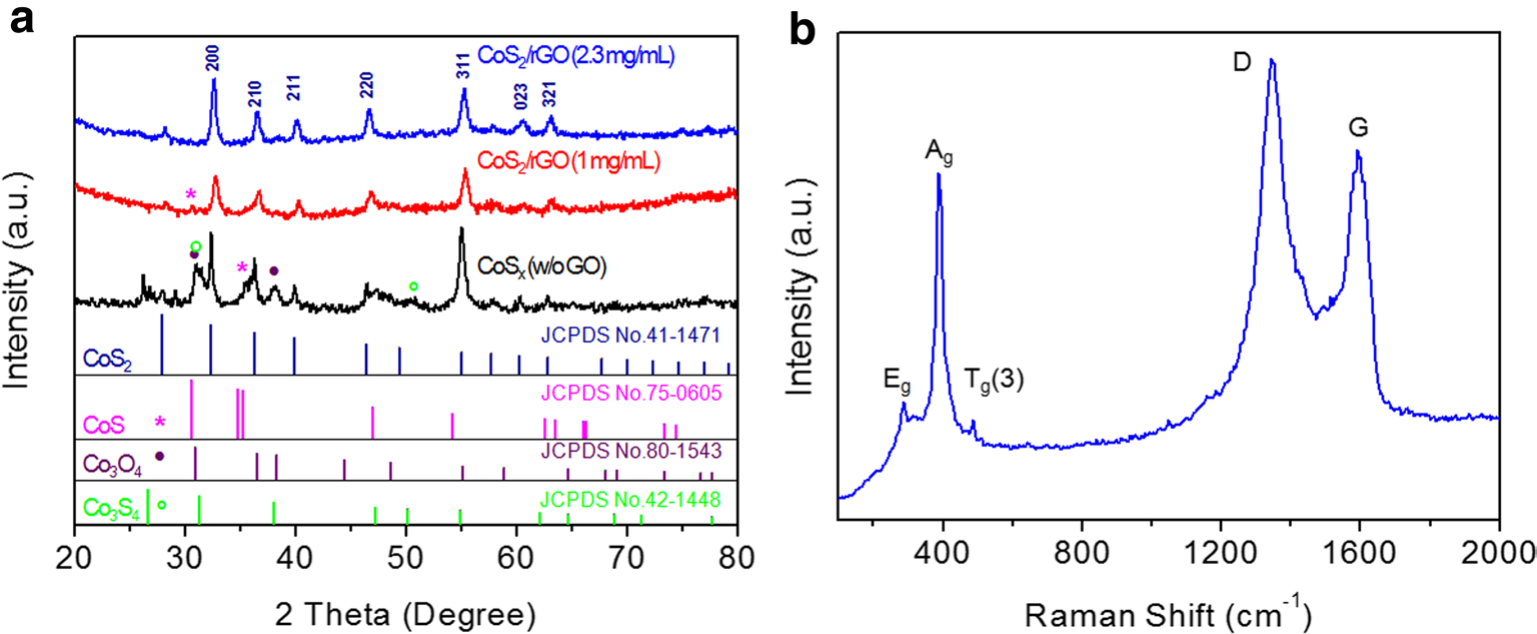
**a** XRD patterns of CoS_2_/rGO. Cobalt sulfides without GO (*black*) and CoS_2_/rGO with 1 mg/mL GO (*red*) and 2.3 mg/mL GO (*blue*) compared with standard patterns for CoS_2_ (*navy lines*), CoS (*pink lines*), Co_3_S_4_ (*green lines*), and Co_3_O_4_ (*violet lines*). **b** Raman spectrum of CoS_2_/rGO with 2.3 mg/mL GO

In a typical hydrothermal reaction with CoS_2_, cobalt precursor such as cobalt chloride reacts with thioacetamide, sodium thiosulfate, etc. to produce CoS_2_ [15–20]. However, the problem with this method involves the generation of impurities such as Co_3_S_4_ and Co_9_S_8_ along with CoS_2_, even though they occur in small proportions [15, 16]. Although there are some reports on the synthesis of CoS_2_/rGO, synthesis of pure CoS_2_ on rGO sheets has not been clearly demonstrated. It should be noted that these preliminary studies employed small amounts of GO, less than 1 mg/mL, in final reactant solutions for hydrothermal reactions [11, 15, 18–21]. Note that growth condition for other reported cobalt sulfide/rGO material is summarized in Additional file 1: Table S1. In this study, we produced pure CoS_2_ on rGO sheets using GO concentrations of greater than 2 mg/mL. Specifically, we carried out hydrothermal reactions at different concentrations of GO (0.2, 1, 1.6, 2, 2.3, 2.6, and 4 mg/mL) in the synthesis of pure CoS_2_ (Table 1). Moreover, cobalt acetate tetrahydrate was used as a precursor for Co because it is known that cobalt acetate yields smaller crystal sizes and higher-BET surfaces than cobalt chloride [22, 23]. Each hydrothermal reaction was carried out at 240 °C for 24 h using cobalt acetate tetrahydrate, thioacetamide (TAA), and GO, followed by a washing of the product with distilled water and drying in vacuum at 60 °C. It is noted that 240 °C was determined to be an optimum temperature from experimental results performed at various temperatures (Additional file 1: Figure S1). For reference, the weight percentage of rGO in some final products is provided in Additional file 1: Table S2. As a control experiment, hydrothermal reaction was also performed without GO, producing a mixture of Co_3_S_4_, CoS, and CoS_2_ (see the XRD data in Fig. 1a below). Interestingly, a hybrid of pure CoS_2_ and rGO (CoS_2_/rGO) was obtained when more than 2 mg/mL of GO was added. It is worth noting that GO is partially reduced during reaction, meaning that the product is CoS_2_/rGO. Actually, Co^2+^ ions can be linked into the functional groups of GO sheets to form strong Co^2+^-linked GO cylinders [24].

**Table 1:** Experimental conditions for CoS_2_/rGO synthesis with various concentrations of GO

Concentration of GO (mg/mL)	Ratio of GO:Co^2+^ (mg:mmol)	Synthesized phase
0	0:1	CoS_2_ + Co_3_S_4_ + Co_3_O_4_ + CoS
0.2	8.8:1	CoS_2_ + Co_3_S_4_ + CoS
1	44:1	CoS_2_ + CoS
1.6	70.4:1	CoS_2_ + CoS
2	88:1	CoS_2_
2.3	101.2:1	CoS_2_
2.6	114.4:1	CoS_2_
4	176:1	CoS_2_

**Fig. 2:**
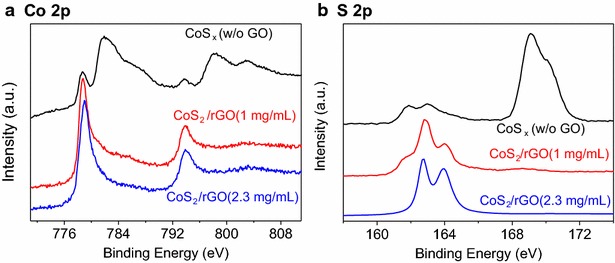
X-ray photoelectron spectra of the **a** Co 2p and **b** S 2p regions for CoS_2_ without GO (*black*) and for CoS_2_/rGO with 1 mg/mL GO (*red*) and 2.3 mg/mL GO (*blue*)

Consequently, it is expected that these cobalt ions are protected from unexpected reaction, meaning that CoS_2_ on rGO is produced, whereas free cobalt ions undergo unwanted reactions during hydrothermal reaction to yield Co_3_S_4_, Co_9_S_8_, and other cobalt sulfides. Furthermore, rGO sheets play a role as an oxidation-resistance layer for cobalt sulfides. CoS_0_ covered by rGO was not oxidized, while Co_3_S_4_ obtained without GO became oxidized to Co_3_O_4_. It is generally known that cobalt sulfides are easily oxidized in air (See the XPS data in Fig. 2 below) [10]. These results are supported by a previous report on the oxidation resistance of multilayer rGO [25]. Since dimeric S_2_^2−^ in VS_4_ can be obtained on graphitic layers, such as those in rGO and carbon nanotubes [26], the formation of pure CoS_2_ on rGO sheets is not unexpected.

**Fig. 3:**
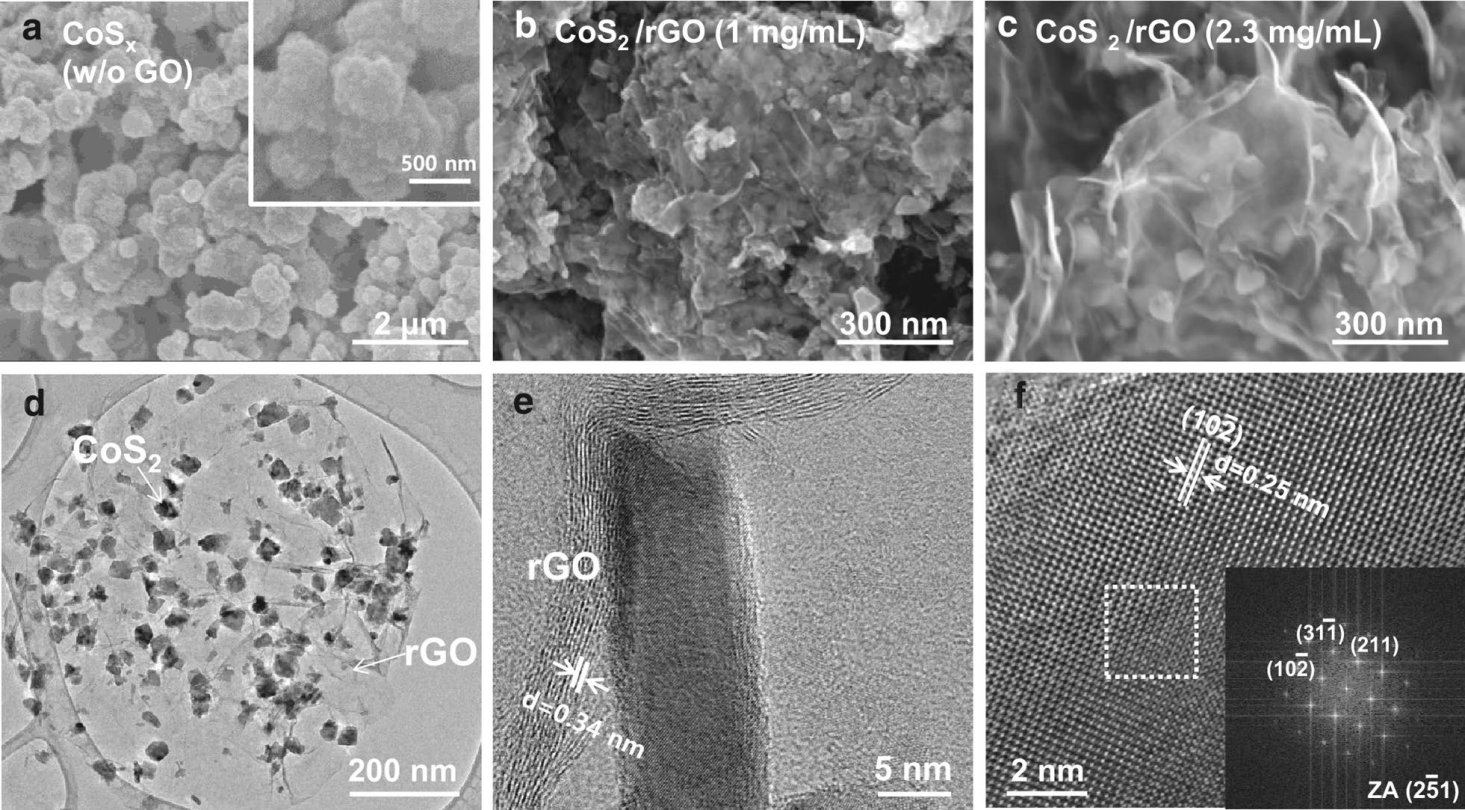
SEM images of CoS_2_ particles **a** without GO, **b** with 1 mg/mL GO, and **c** with 2.3 mg/mL GO. **d** Low magnification TEM image of CoS_2_/rGO with 2.3 mg/mL GO. **e** An enlarged TEM image of CoS_2_/rGO and **f** a high resolution TEM image of CoS_2_, the latter showing d-spacing of 0.27 nm in the (200) plane. The *inset* in **f** provides a diffraction pattern of the CoS_2_ in the *white square*

Scanning electron microscopy (SEM) images of CoS_2_/rGO with different concentrations of GO and a mixture of cobalt sulfides without GO are shown in Fig. 3. In the absence of GO, an aggregation of particles occurred (Fig. 3a and the TEM images in Additional file 1: Figure S2). On the other hand, CoS_2_ nanoparticles were covered by the rGO sheets for GO concentrations of 1 and 2.3 mg/mL (Fig. 3b, c). The transmission electron microscopy (TEM) images also demonstrate the growth of single-crystalline CoS_2_ nanoparticles on the rGO sheets (Fig. 3d, e). Figure 3e shows the rGO sheets with an interlayer d-spacing of 0.34 nm, which corresponds to the d-spacing of graphite, surrounding the CoS_2_. Figure 3f shows CoS_2_ nanoparticles with a d-spacing of 0.25 nm, which corresponds to the inter-planar spacing of the (210) plane of the cubic phase. In addition, the SAED pattern establishes the single-crystalline nature of the CoS_2_ particles (inset of Fig. 3f). To identify the stoichiometry of Co and S, an elemental mapping of the CoS_2_/rGO hybrids was performed via energy-dispersive X-ray spectroscopy (EDX); this mapping indicated that the ratio of S to Co was 2 (Additional file 1: Figure S3). Note that the C and O in the EDX spectrum are attributed to the rGO.

Figure 1a shows the X-ray diffraction (XRD) spectra of the products according to GO concentration. When GO is not added, there are not only reflections of CoS_2_ such as the (200), (220), and (311) planes, but also reflections for CoS, Co_3_S_4_, and Co_3_O_4_. This result is in good agreement with previous reports stating that multiphase cobalt sulfides are usually synthesized in these conditions [15, 16]. Note that CoS was partially observed along with CoS_2_ for a 1 mg/mL GO concentration (Fig. 1a, JCPDS 75-0605). This result is consistent with the TEM results, which indicate a d-spacing corresponding to the (100) plane of hexagonal-phase CoS within the CoS_2_/rGO products (Additional file 1: Figure S4, space group P63/mmc; a = 3.384 Å, c = 5.16 Å). In addition, our XRD pattern for CoS_2_/rGO did not show signs of oxidation, while the non-GO Co_3_S_4_ showed a pattern for Co_3_O_4_ (Fig. 1a, JCPDS 42-1448; JCPDS 80-1543). Furthermore, we calculated the average crystal size by Scherrer formula using the (200) plane. The results show 36.65 nm and 33.82 nm for the CoS_2_/rGO (2.3 mg/mL) and CoS_2_/rGO (1 mg/mL), respectively. These results are well matched with the SEM data in Fig. 3. Note that XRD spectra for CoS_2_/rGO samples with various GO concentrations in Table 1 are shown in Additional file 1: Figure S5 and pure-phase CoS_2_ was obtained when GO concentration is higher than 2 mg/mL. A representative Raman spectrum of CoS_2_/rGO (2.3 mg/mL of GO) is shown in Fig. 1b. Characteristic peaks for CoS_2_ at 288, 388, and 484 cm^−1^ corresponding to the E_g_, A_g_, and T_g_(3) modes, respectively [27], and the D and G bands of rGO, can be clearly seen.

To verify the oxidation states of the elements in the CoS_2_/rGO products, we measured their X-ray photoelectron spectroscopy (XPS) spectra (Fig. 2). The peaks for the binding energy of Co 2p_3/2−1/2_ appeared at 778.8 and 794 eV for the CoS_2_/rGO sample with 1 mg/mL GO and at 779 and 794.2 eV for the sample with 2.3 mg/mL GO, indicating an oxidation state of Co^2+^ (Fig. 2a) [10]. The peaks around 786 and 803 eV are associated with the shake-up type peaks of the 2p_3/2-1/2_ and peaks at 782.3 and 798 eV are associated with Co^3+^ from the mixture of cobalt sulfides without GO [28]. For the case of S 2p_3/2-1/2_, doublet peaks appear at 162.9 and 164 eV for the CoS_2_/rGO with 1 mg/mL GO sample and at 162.6 and 163.9 eV for the CoS_2_/rGO with 2.3 mg/mL GO sample, indicating the presence of S_2_^2−^ (Fig. 2b) [10]. Note that there is an additional peak at ~161.8 eV for S^2−^ in the CoS_2_/rGO with 1 mg/mL GO sample and cobalt sulfides without rGO [29]. Although CoS_2_ is easily oxidized in air as mentioned above [10], our results show no peak for oxidation in any of the CoS_2_/rGO samples, while there is a substantial oxidation peak at 169.1 eV for the non-GO sample in Fig. 2b, which is consistent with the XRD spectra. Consequently, the XPS results confirm the pure-phase nature of the CoS_2_ for GO concentration of 2.3 mg/mL.

**Fig. 4:**
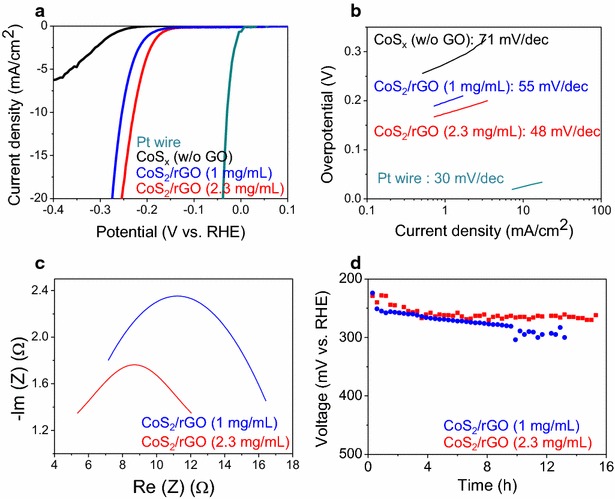
Catalytic activity for hydrogen evolution reactions. **a** Polarization curves (iR-corrected) of Pt wires (*green*), the mixture of cobalt sulfide without GO (*black*), and CoS_2_/rGO samples with 1 mg/mL GO (*blue*) and with 2.3 mg/mL GO (*red*). **b** Tafel analysis of the data shown in **a**. **c** Electrochemical impedance spectroscopy (EIS) for CoS_2_/rGO samples. **d** Long-term stability measurements for CoS_2_/rGO samples

To explore practical viability, HER tests with the CoS_2_/rGO samples on glassy carbon electrodes were carried out using a three-electrode cell with a 0.5 M sulfuric acid electrolyte. Polarization curves (I–V plots) were taken for CoS_2_/rGO samples (1 and 2.3 mg/mL GO) along with a mixture of cobalt sulfides without GO (Fig. 4a). The overpotential of the 2.3 and 1 mg/mL GO samples were found to be −150 and −180 mV versus RHE, respectively. The overpotentials required to drive current densities of 10 mA/cm^2^ were 228 mV for the 2.3 mg/mL GO sample and 250 mV for the 1 mg/mL GO sample. The mixture of cobalt sulfides without GO exhibited HER activity with an overpotential of −280 mV versus RHE. The Tafel plots derived from these data are shown in Fig. 4b wherein the linear portions were fitted to the Tafel equation to determine the slopes. The Tafel plots reveal a slope of 71 mV/dec for the mixture of cobalt sulfides without GO, 55 mV/dec for CoS_2_/rGO with 1 mg/mL GO, and 48 mV/dec for CoS_2_/rGO with 2.3 mg/mL GO. The lower Tafel slope value for the CoS_2_/rGO hybrid materials, compared to the non-GO sample, can be attributed to the contribution of CoS_2_ as an active material and the presence of conductive rGO. The pure-phase catalyst clearly plays a key role in revealing the intrinsic activity of CoS_2_, and the metallic property of CoS_2_ allows efficient charge transport from the electrode to the surface of the catalysts, which is desired for high-performance electrocatalysts [10]. It should also be noted that the impedance results indicate a lower resistance in the CoS_2_/rGO electrode with 2.3 mg/mL GO than with 1 mg/mL GO (Fig. 4c). Thus, the formation of an interconnected conducting rGO network can facilitate rapid electron transfer from the electrode to the catalyst. This tendency that the CoS_2_/rGO electrode with 2.3 mg/mL GO showed higher catalytic activity than that with 1 mg/mL GO was also confirmed in the capacitive current for each CoS_2_ electrode as a function of scan rate which reveals the double layer capacitance (Additional file 1: Figure S6). The HER performance for the CoS_2_/rGO samples with various concentrations of GO was also measured (Additional file 1: Figure S7). Considering the onset potentials and Tafel slopes, we found that 2 and 2.3 mg/mL represent optimized concentrations of GO in terms of HER performance. With concentrations lower than 2.0 mg/mL, CoS is partially included and resistance is relatively high. With 4 mg/mL, rGO is expected to hinder the exposure of CoS_2_ as a catalyst, and the charge-transfer resistance of the CoS_2_/rGO sample increased slightly (Additional file 1: Figure S8). Recently, the performance of HER for CoS_2_ film (overpotential of −180 mV and Tafel slope of 44.6 mV/dec) was reported [3]. Thus, the onset potential and Tafel slope values of CoS2/rGO (2.3 mg/mL GO) are roughly commensurate with those of the CoS_2_ film. However, our catalysts have an improved a long-term stability than that reported for the CoS_2_ film, which is relatively limited. That is, CoS_2_ films are known to become delaminated with a physical loss of catalysts occurring within 1 h during cycling. However, CoS_2_/rGO electrodes maintain hydrogen evolution at J_cathodic_ = 10 mA/cm^2^ with minimal change in the applied overpotential, as shown in Fig. 4d. It is noted that the phase and structure of CoS_2_/rGO maintained after the cycling for 14 h (Additional file 1: Figure S9). After 15 h, the catalysts became delaminated. We summarize the HER performance for various CoS_2_ structures in the literature, including our results in Additional file 1: Table S3.

## Conclusions

In summary, we successfully synthesized CoS_2_/rGO with pure-phase CoS_2_ on rGO sheets via hydrothermal reaction. The CoS_2_/rGO hybrid materials exhibited high catalytic activity for HER: overpotential of −150 mV versus RHE and a Tafel slope of 48 mV/dec for CoS_2_/rGO (2.3 mg/mL GO). Thus, the present study demonstrates the large-scale synthesis of CoS_2_/rGO hybrids with a long-term stability and strong HER performance.

## Additional file

**Additional file 1: Figure S1.** XRD patterns of CoS /rGO samples synthesized at different temperatures: 200 (black), 240 (red),and 265 C (blue). **Figure S2.** TEM image of cobalt sulfides without GO: (a) low magnification and (b) high-magnificationimages. The inset in (b) indicates the (111) diffraction pattern of Co S in the white square. **Figure S3.** EDS elemental mapping of Co and S for CoS /rGO (2.3 mg/mL of GO), EDX spectrum and tableshowing elemental composition. The atomic ratio of S and Co is 2, confirming the stoichiometry of CoS . **Figure S4.** TEM image of CoS /rGO (1 mg/mL of GO). (a) Bright field TEM image in low magnification (b)High resolution TEM image of CoS particle. (c) High resolution TEM image of a CoS particle which is not covered by rGO. Inset of (b) shows planes of CoS including (311) plane and inset of (c) shows planes of CoS including (100) plane. **Figure S5.** XRD patterns of the CoS /rGO hybrids with various GO concentrations. **Figure S6.** (a) Plots for the extraction of the double-layer capacitance (C ) for CoS /rGO electrodes. CV cycles of CoS /rGO samples with 1 mg/mL of GO (b) and 2.3 mg/mL of GO (c) at different scan rates. **Figure S7.** Polarization curves of CoS /rGO depending on the concentration of GO at (a) higher and (b) lower applied oveportentials show the HER performance. (c) Tafel analysis of the data shown in Figure S5a. (d) Summary of the electrochemical performance for CoS /rGO samples with various GO concentrations. **Figure S8.** Electrochemical impedance spectroscopy (EIS) for CoS /rGO samples. **Figure S9.** (a) SEM image of CoS /rGO (2.3 mg/mL of GO) after the cycling test for 14 hours. (b) High resolution TEM image of a CoS particle after the cycling test. Inset of (b) shows planes of CoS including (102) plane. **Table S1.** Comparison of growth condition for CoS /rGO with other reported cobalt sulfide/rGO materials. **Table S2.** The weight percentage of rGO in products by elemental analysis. **Table S3.** Comparison of HER activity measured for our CoS2/rGO with other reported CoS materials as HER catalyst.
