# Supplementary material for: Selective synthesis of pure cobalt disulfide on reduced graphene oxide sheets and its high electrocatalytic activity for hydrogen evolution reaction
Source: Nano Converg. 2016 Jan 27;3:5. doi: 10.1186/s40580-016-0066-x (PMC5271118; doi:10.1186/s40580-016-0066-x)
Supplement: Supplementary file 1 — Additional file 1: Figure S1. XRD patterns of CoS /rGO samples synthesized at different temperatures: 200 (black), 240 (red),and 265 C (blue). Figure S2. TEM image of cobalt sulfides without GO: (a) low magnification and (b) high-magnificationimages. The inset in (b) indicates the (111) diffraction pattern of Co S in the white square. Figure S3. EDS elemental mapping of Co and S for CoS /rGO (2.3 mg/mL of GO), EDX spectrum and tableshowing elemental composition. The atomic ratio of S and Co is 2, confirming the stoichiometry of CoS . Figure S4. TEM image of CoS /rGO (1 mg/mL of GO). (a) Bright field TEM image in low magnification (b)High resolution TEM image of CoS particle. (c) High resolution TEM image of a CoS particle which is not covered by rGO. Inset of (b) shows planes of CoS including (311) plane and inset of (c) shows planes of CoS including (100) plane. Figure S5. XRD patterns of the CoS /rGO hybrids with various GO concentrations. Figure S6. (a) Plots for the extraction of the double-layer capacitance (C ) for CoS /rGO electrodes. CV cycles of CoS /rGO samples with 1 mg/mL of GO (b) and 2.3 mg/mL of GO (c) at different scan rates. Figure S7. Polarization curves of CoS /rGO depending on the concentration of GO at (a) higher and (b) lower applied oveportentials show the HER performance. (c) Tafel analysis of the data shown in Figure S5a. (d) Summary of the electrochemical performance for CoS /rGO samples with various GO concentrations. Figure S8. Electrochemical impedance spectroscopy (EIS) for CoS /rGO samples. Figure S9. (a) SEM image of CoS /rGO (2.3 mg/mL of GO) after the cycling test for 14 hours. (b) High resolution TEM image of a CoS particle after the cycling test. Inset of (b) shows planes of CoS including (102) plane. Table S1. Comparison of growth condition for CoS /rGO with other reported cobalt sulfide/rGO materials. Table S2. The weight percentage of rGO in products by elemental analysis. Table S3. Comparison of HER activity measured for our C [file 40580_2016_66_MOESM1_ESM.docx]

***Supporting Information***

**Selective Synthesis of Pure Cobalt Disulfide on Reduced Graphene Oxide Sheets and Its High Electrocatalytic Activity for Hydrogen Evolution Reaction**

Seongjoon Ahn^‡2,3^, Jieun Yang^‡5^, Hyunseob Lim^1,3,4^ and Hyeon Suk Shin^1,2,3,4,*^

^1^Department of Chemistry, Ulsan National Institute of Science and Technology (UNIST), UNIST-gil 50, Ulsan 689-798, Republic of Korea Tel: +82-52-17-81022; E-mail: shin@unist.ac.kr Address here.

^2^Department of Energy Engineering, Ulsan National Institute of Science and Technology (UNIST), UNIST-gil 50, Ulsan 689-798, Republic of Korea

^3^Low Dimensional Carbon Materials, Ulsan National Institute of Science and Technology (UNIST), UNIST-gil 50, Ulsan 689-798, Republic of Korea

^4^Center for Multidimensional Carbon Materials, Institute of Basic Science, Ulsan National Institute of Science and Technology (UNIST), UNIST-gil 50, Ulsan 689-798, Republic of Korea

^5^Department of Material Science & Engineering, Rutgers University, Piscataway, New Jersey 08854, United States

‡These authors equally contributed to this work.

^*^To whom correspondence should be addressed, E-mail: shin@unist.ac.kr

**Figure S1**. XRD patterns of CoS_2_/rGO samples synthesized at different temperatures: 200 (black), 240 (red), and 265 ^o^C (blue).

**Figure S2**. TEM image of cobalt sulfides without GO: (a) low magnification and (b) high-magnification images. The inset in (b) indicates the (111) diffraction pattern of Co_3_S_4_ in the white square.

**Figure S3**. EDS elemental mapping of Co and S for CoS_2_/rGO (2.3 mg/mL of GO), EDX spectrum and table showing elemental composition. The atomic ratio of S and Co is 2, confirming the stoichiometry of CoS_2_.

**Figure S4**. TEM image of CoS_2_/rGO (1 mg/mL of GO). (a) Bright field TEM image in low magnification (b) High resolution TEM image of CoS_2_ particle. (c) High resolution TEM image of a CoS particle which is not covered by rGO. Inset of (b) shows planes of CoS_2_ including (311) plane and inset of (c) shows planes of CoS including (100) plane.

**Figure S5**. XRD patterns of the CoS_2_/rGO hybrids with various GO concentrations.

**Figure S6**. (a) Plots for the extraction of the double-layer capacitance (C_dl_) for CoS_2_/rGO electrodes. CV cycles of CoS_2_/rGO samples with 1 mg/mL of GO (b) and 2.3 mg/mL of GO (c) at different scan rates.

**
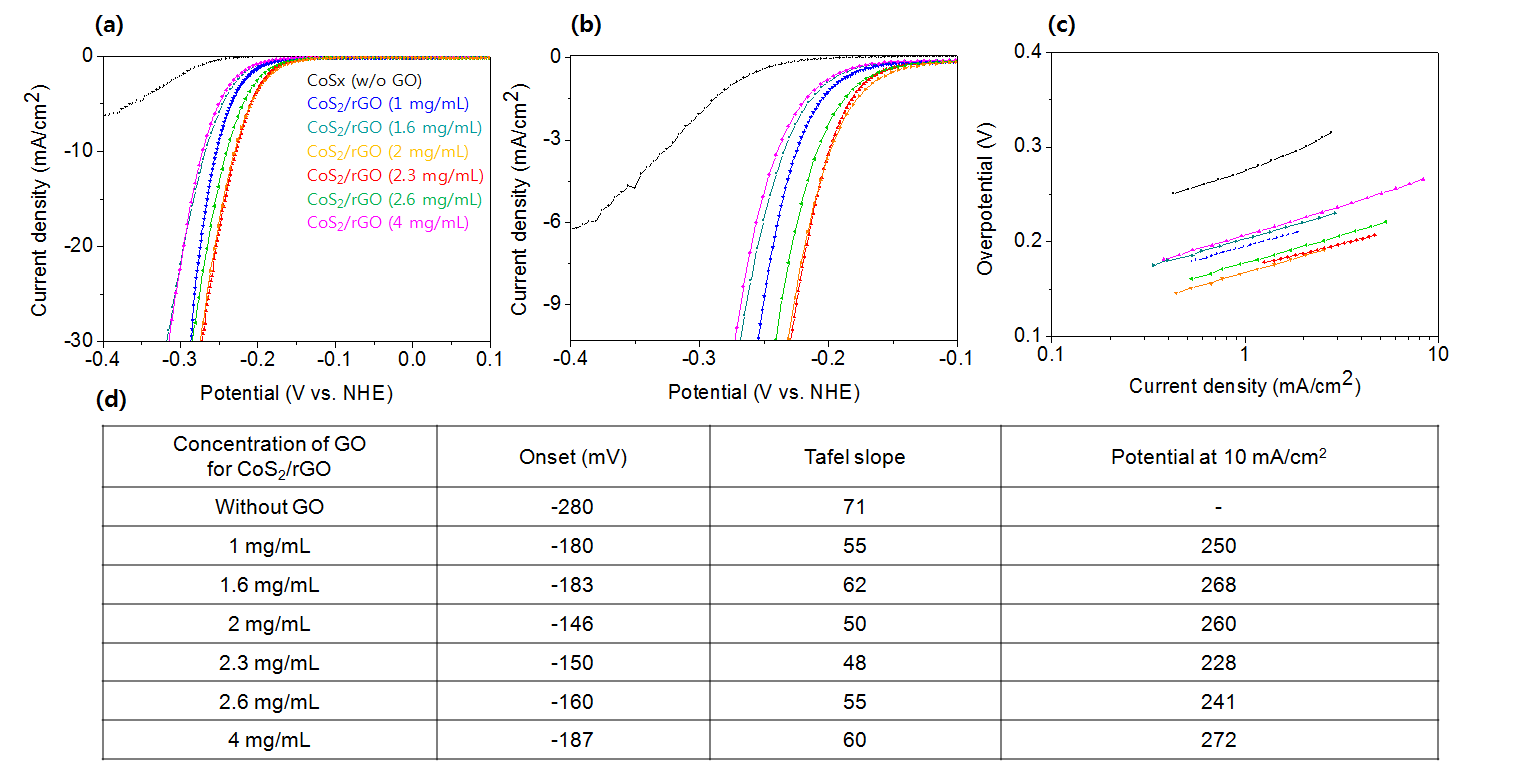
**

**Figure S7**. Polarization curves of CoS_2_/rGO depending on the concentration of GO at (a) higher and (b) lower applied oveportentials show the HER performance. (c) Tafel analysis of the data shown in **Figure S5a**. (d) Summary of the electrochemical performance for CoS_2_/rGO samples with various GO concentrations.

Figure S8. Electrochemical impedance spectroscopy (EIS) for CoS_2_/rGO samples.

**Figure S9**. (a) SEM image of CoS_2_/rGO (2.3 mg/mL of GO) after the cycling test for 14 hours. (b) High resolution TEM image of a CoS_2_ particle after the cycling test. Inset of (b) shows planes of CoS_2_ including (102) plane.

**Table S1.** Comparison of growth condition for CoS_2_/rGO with other reported cobalt sulfide/rGO materials

| **Amount of GO** | **Total volume** | **Concentration of GO** | **Ratio of**  **GO : Co^2+^**  **(mg : mmol)** | **Product** | **Reference** |
| --- | --- | --- | --- | --- | --- |
| 1.5 mg | 10 mL H_2_O | 0.15 mg/mL | 18.75 : 1 | Co_1-x_S/rGO | [11] |
|  | 80 mL EtOH | 0.3 mg/mL | 12 : 1  (Assume that GO 24 mg) | CoS_2_/rGO | [16] |
| 100 mg | 200 mL H_2_O | 0.5 mg/mL | 100 : 1 | CoS_2_/rGO | [18] |
| 50 mg | 50 mL H_2_O + 20 mL precursor solution | 0.714 mg/mL | 62.5 : 1 | CoS_2_/rGO | [19] |
|  | 20 mL H_2_O | < 1 mg/mL | 8 : 1  (Assume that GO 20 mg) | CoS_2_/rGO | [20] |
| 800 mg | 400 mL H_2_O | 2 mg/mL | 88 : 1 | CoS_2_/rGO | Our result |

**Table S2**. The weight percentage of rGO in products by elemental analysis.

|  | Concentration of GO | | | |
| --- | --- | --- | --- | --- |
|  | 1 mg/mL | 2 mg/mL | 2.3 mg/mL | 4 mg/mL |
| Wt% of rGO | 27.8 | 40.2 | 41.3 | 51.2 |

**Table S3.** Comparison of HER activity measured for our CoS_2_/rGO with other reported CoS_2_ materials as HER catalyst

| **Sample** | **Onset potential**  **(mV)** | **Tafel slope**  **(mV/dec)** | **Long-term stability** | **Exchange current density (A/cm^2^)** | **Reference** |
| --- | --- | --- | --- | --- | --- |
| CoS_2_ film | −180 | 44.6 | — | 5.4 x 10^-8^ | [3] |
| CoS_2_ film | −170 | 51.4 | <5 hours | 1.97 x 10^-6^ | [10] |
| CoS_2_ MW | −100 | 58.0 | 41 hours | 5.27 x 10^-6^ |  |
| CoS_2_ NW | −75 | 51.6 | 5 hours | 2.80 x 10^-6^ |  |
| CoS_2_/rGO | −300 | 82 | — | 7.02 x 10^-6^ | [21] |
| CoS_2_/rGO | −150 | 48 | 15 hours | 1.4 x 10^-6^ | Our result |
